# Wnt/β-Catenin Signaling as a Potential Target for the Treatment of Liver Cirrhosis Using Antifibrotic Drugs

**DOI:** 10.3390/ijms19103103

**Published:** 2018-10-10

**Authors:** Koji Nishikawa, Yosuke Osawa, Kiminori Kimura

**Affiliations:** 1 Department of Hepatology, Tokyo Metropolitan Cancer and Infectious Diseases Center Komagome Hospital, 3-18-22 Honkomagome, Bunkyo-ku, Tokyo 113-8677, Japan; k-nishi456@cick.jp; 2 The Research Center for Hepatitis and Immunology, National Center for Global Health and Medicine, 1-7-1 Kohnodai, Ichikawa, Chiba 272-8516, Japan; osawa-gif@umin.ac.jp

**Keywords:** liver fibrosis, Wnt, β-catenin, PRI-724

## Abstract

Cirrhosis is a form of liver fibrosis resulting from chronic hepatitis and caused by various liver diseases, including viral hepatitis, alcoholic liver damage, nonalcoholic steatohepatitis, and autoimmune liver disease. Cirrhosis leads to various complications, resulting in poor prognoses; therefore, it is important to develop novel antifibrotic therapies to counter liver cirrhosis. Wnt/β-catenin signaling is associated with the development of tissue fibrosis, making it a major therapeutic target for treating liver fibrosis. In this review, we present recent insights into the correlation between Wnt/β-catenin signaling and liver fibrosis and discuss the antifibrotic effects of the cAMP-response element binding protein/β-catenin inhibitor PRI-724.

## 1. Introduction

Liver fibrosis develops as a result of chronic hepatitis caused by various factors, including hepatitis virus infection, excessive alcohol use, and metabolic and autoimmune disorders [1,2]. Conditions that result in continuous inflammation lead to the accelerated production and deposition of extracellular matrix (ECM) components and increased fibrosis [3]. Therefore, while an acute liver injury might recover completely, chronic, long-term liver disease often leads to fibrosis progressing to cirrhosis. Liver cirrhosis causes hepatocyte dysfunction and portal hypertension, resulting in further complications, such as esophageal varices, ascites, hepatic encephalopathy, hepatocellular carcinoma [4], and eventually, a decreased rate of survival. Patients with compensated or decompensated cirrhosis have a 4.7- or 9.7-fold higher risk of mortality, respectively, as compared with healthy controls [5].

The most prevalent cause of liver fibrosis is infection with the hepatitis C virus (HCV) [6]. Currently, the risks posed by continuous HCV infection have been almost completely eliminated due to the development of direct-acting antivirals. There are contradictory reports concerning the effect that curing HCV has on fibrosis progression. Although some studies show that curing HCV can improve the symptoms of liver fibrosis [7], others report that liver fibrosis can progress, even after eradication of HCV [8]. Additionally, resolution of liver fibrosis after HCV elimination requires a prolonged period of time, indicating that curing HCV might not be the optimal approach to countering fibrosis. Therefore, it is important to develop drugs capable of directly treating fibrosis [9,10,11].

The Wnt/β-catenin pathway is associated with the regulation of cell proliferation and differentiation [12,13], embryonic development, and maintenance of homeostasis and is linked to many human diseases [14]. Activated Wnt/β-catenin signaling has been implicated in fibrosis in a number of organ systems, including the lung, kidney, skin, and liver [15,16]. Modulation of the small molecule Wnt has proven to be extremely effective in countering fibrosis in murine models of lung, kidney, and liver fibrosis [17,18]. Additionally, antifibrotic effects of Wnt/β-catenin-signaling blockage have been reported, with specific inhibition of cAMP response element binding protein (CBP)/β-catenin interactions by ICG-001 attenuating bleomycin-induced lung fibrosis in mice [18]. Moreover, ICG-001 also ameliorates renal intestinal fibrosis induced by unilateral ureteral obstruction in mice [19] and reverses late-stage fibrotic injuries in murine models [18,19,20]. A recent study reported that a selective inhibitor of CBP/β-catenin interaction (PRI-724) exhibited antifibrotic effects in liver fibrosis murine models by attenuating liver fibrosis via inhibiting hepatic stellate cell (HSC) activation and promoting macrophage-mediated inflammation resolution [21]. These findings suggest that PRI-724 has therapeutic potential for the treatment of liver fibrosis. This review focuses on correlations between the Wnt/β-catenin pathway and liver fibrosis, as well as the effects of PRI-724 against liver fibrosis.

## 2. Mechanisms of Liver Fibrosis

Chronic tissue injury leads to fibrosis in many organs, including the liver, lungs, kidneys, and heart. In chronic liver disease and irrespective of the underlying etiology, the development of fibrosis is the first step toward in the progression to cirrhosis. Although many factors have been characterized as mediators of liver fibrosis, the underlying molecular mechanism remains poorly defined, and there is currently no effective therapy. HSCs play a key role in liver fibrosis [1,22]. In the healthy liver, HSCs localize in the space of Disse, store vitamin A, and represent quiescent HSCs [3,23]. Injured hepatocytes, endothelial cells, and bile-duct cells release damage-associated molecular patterns (DAMPs) and reactive oxygen species (ROS), which can directly activate HSCs. Viral proteins can also directly contribute to HSC activation, with direct HCV infection of HSCs previously reported [24]. Inflammatory cells, such as Kupffer cells, T lymphocytes, and neutrophils, are recruited to the damaged site and activated by DAMPs and ROS in order to secrete proinflammatory factors [25,26], including cytokines (e.g., interleukin (IL)-1β and IL-6), chemokines (e.g., C–C chemokine ligand 2 (CCL2)), growth factors (e.g., transforming growth factor-β (TGF-β) and platelet-derived growth factor), and tumor necrosis factor (TNF) [27]. These profibrotic factors affect quiescent HSCs both directly and indirectly. HSCs undergo an activation process and change their phenotype from quiescent retinoid-storing HSCs to collagen-producing contractile myofibroblasts expressing α-smooth muscle actin (αSMA) [28] through activation of the mitogen-activated protein kinase and Smad-signaling pathways [29]. ECM components, such as collagen, fibronectin, elastin, laminin, and proteoglycan, are produced excessively by activated HSCs and accumulate in the liver. Wnt signaling is stimulated in activated HSCs, and inhibition of Wnt signaling by adenoviral transduction of the Wnt co-receptor antagonist Dickkopf-1 restores HSC quiescence and increases apoptosis in cultured HSCs [30]. An inhibitor of the CBP/β-catenin interaction (C-82) inhibits activation of primary cultured mouse HSCs [21], indicating that β-catenin-mediated signals are involved in HSC activation.

**Figure 1:**
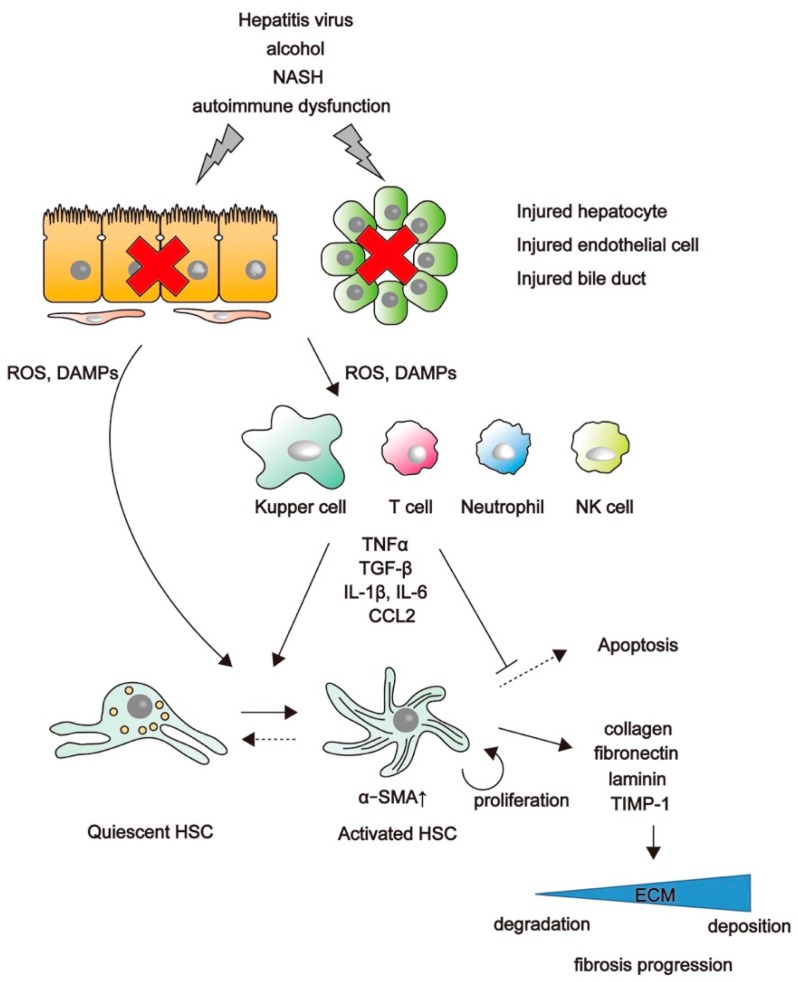
Process of hepatic stellate cell (HSC) activation and liver fibrosis progression. Damaged cells secrete damage-associated molecular patterns (DAMPs) and reactive oxygen species (ROS). These factors activate quiescent HSCs directly and indirectly through the action of Kupffer cells, T cells, neutrophils, and natural killer (NK) cells. Activated HSCs produce extracellular matrix (ECM) components and tissue inhibitors of metalloproteinases (TIMPs), which accelerates the deposition of ECM components and results in liver fibrosis progression. CCL2, C–C chemokine ligand 2; IL, interleukin; NASH, nonalcoholic steatohepatitis; SMA, smooth muscle actin; TNF, tumor necrosis factor.

The contribution of macrophages to liver fibrosis has also been reported [31,32]. Macrophages can perform both injury inducing and repair-promoting roles simultaneously in an injured organ. The depletion of liver macrophages aggravates hepatocellular damage while suppressing liver fibrosis following bile-duct ligation [33]. By contrast, macrophage depletion during fibrosis resolution leads to the failure of matrix degradation [34], suggesting that hepatic macrophages are also involved in the regression of hepatic fibrosis. Additionally, a role for β-catenin in macrophages has been reported, with macrophage-specific knockdown of β-catenin causing insufficient skin-wound healing due to defects in migration, adhesion to fibroblasts, and TGF-β production [35]. The balance between matrix metalloproteinases (MMPs), which affect ECM degradation, and tissue inhibitors of MMPs (TIMPs), is also closely related to liver fibrosis [36]. In the regression phase of liver fibrosis, intrahepatic MMPs are expressed prior to TIMPs, which are prominent during the formation of scar tissue [37]. Rapid treatment or removal of the conditions leading to hepatitis possibly promote apoptosis or inactivation of activated HSCs, with this decrease in activated HSCs reducing TIMP expression, which increases MMP activity and results in degradation of accumulated ECM components. However, chronic hepatitis permanently activates HSCs and causes dominant expression of TIMPs. In a model of murine liver fibrosis induced by carbon tetrachloride (CCl_4_), administration of PRI-724 (the inhibitor of CBP/β-catenin interaction) accelerated the resolution of liver fibrosis accompanied by an increase in MMP-9, MMP-2, and MMP-8 levels in intrahepatic leukocytes [21]. Macrophages are also associated with the balance of TIMP and MMP levels. In a murine model, the activation of Kupffer cells and Ly-6C^high^ macrophages activates HSCs during the progression of liver fibrosis, whereas Ly-6C^low^ macrophages are recruited to produce MMPs during the regression phase (Figure 1) [38]. Therefore, β-catenin-mediated signals are not only associated with liver fibrogenesis but also with the resolution of liver fibrosis through regulating the balance between MMP and TIMP levels.

## 3. Involvement of the Wnt/β-Catenin Pathway in Liver Fibrosis

### 3.1. The Wnt/β-Catenin Pathway

**Figure 2:**
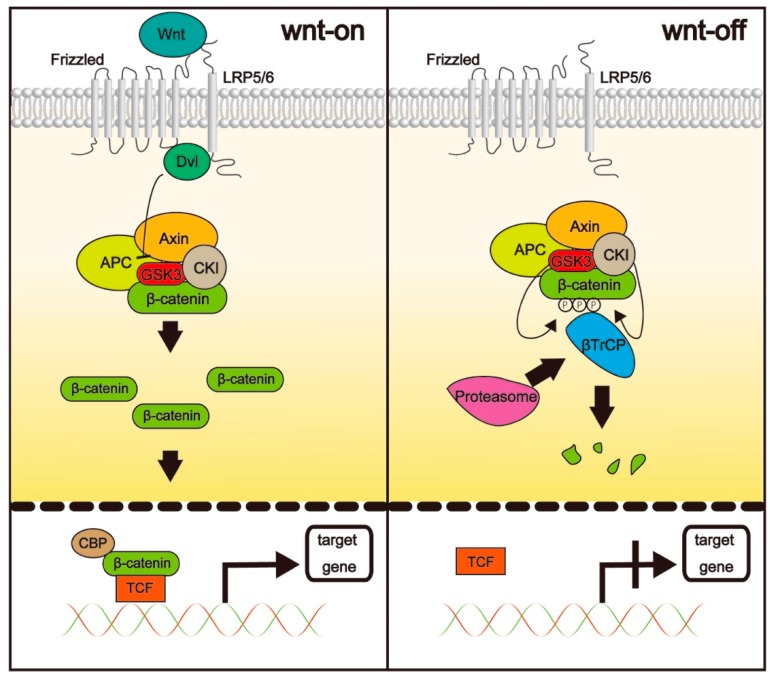
In the presence of Wnt, Dishevelled (Dvl) prevents β-catenin phosphorylation by inhibiting glycogen synthase kinase 3β (GSK3β) activity. β-catenin is stabilized and migrates to the nucleus, where it binds to T cell factor (TCF). In the absence of Wnt signaling, β-catenin is phosphorylated by GSK3β and casein kinase 1α (CK1α) and subsequently ubiquitinated by β-transducin repeat containing protein (βTrCP). Finally, β-catenin is degraded by the proteasome.

β-catenin is a protein with dual functions, acting as both an adhesion molecule and a transcription factor [39]. The functions of β-catenin as a transcription factor are mainly regulated by Wnt proteins, which are cysteine-rich glycoproteins with a molecular weight of ~40 kDa and generally secreted to the ECM [40]. β-catenin is usually found in the cytoplasm, where its stability is controlled by a destruction complex comprising the tumor-suppressor scaffold protein axin, adenomatous polyposis coli, and serine-threonine kinases, such as glycogen synthase kinase 3β (GSK3β) and casein kinase 1α (CK1α). When canonical Wnt/β-catenin signaling is inactive, β-catenin concentration is kept low via its degradation by the destruction complex. In the absence of Wnt signaling, β-catenin is phosphorylated by GSK3β and CK1α. β-Transducin repeat-containing protein ubiquitinates phosphorylated β-catenin, promoting its subsequent proteasomal degradation. When Wnt signaling is active, Wnt binds the seven-transmembrane domain receptor Frizzled (FZD) and the single-transmembrane-domain receptor low-density lipoprotein-receptor-related protein (LRP)5/6 to form a heterotrimer (Wnt-FZD-LPR5/6), which inhibits β-catenin phosphorylation through recruitment of Dishevelled, which inhibits GSK3β. As a consequence, the proportion of unphosphorylated β-catenin increases, followed by its translocation to the nucleus, where it binds to T cell factor (TCF), a DNA-binding protein that interacts with histone acetyltransferase Kat3 family members, CBP, and p300 [41]. The TCF/β-catenin complex binds DNA and promotes the transcription of Wnt target genes, such as *Cyclin D1* [42], *c-Myc* [43], *Axin-2* [44], and *c-Jun* [45] (Figure 2).

Wnt/β-catenin signaling is necessary for organismal development, as evidenced by the embryonic lethality resulting from a defect in gastrulation in mice lacking β-catenin [46]. Wnt/β-catenin signaling is also important for postnatal liver development. Mice with conditional loss of β-catenin in hepatocytes reportedly display a significant decrease in the liver weight:body weight ratio [16]. Similarly, mice with hepatocyte-specific deletion of LRP5 and LRP6 [47], as well as those with hepatocyte-specific deletion of leucine-rich repeat-containing G protein-coupled receptor (LGR)4 and LGR5 (regulators of Wnt signaling) [48], exhibited significantly reduced liver weight. Recently, the Wnt/β-catenin pathway was associated with organ fibrosis [49,50], suggesting that it might represent a new therapeutic target for liver fibrosis [30]. Additionally, Wnt/β-catenin signaling is implicated in HSC activation, as conditional deletion of β-catenin in the mesenchyme during liver development leads to increased expression of αSMA in HSCs and increased collagen deposition in the developing liver [51]. Moreover, mRNA levels of canonical (Wnt3a and 10b) and noncanonical (Wnt4 and 5a) Wnt genes, the Frizzled (FZD) precursors Fz-1 and -2, and co-receptors [LRP6 and Ryk] are increased in culture-activated HSCs relative to levels in quiescent HSCs [30]. Nuclear β-catenin levels and TCF DNA-binding are also markedly increased in activated HSCs. Although Wnt signaling is upregulated in activated HSCs but not in quiescent cells [30], a study showed that β-catenin-dependent canonical Wnt signaling is active in quiescent HSCs, and that treatment with TWS119, a GSK3β inhibitor, impeded synthesis of αSMA [52]. These findings indicate that Wnt signaling maintains the quiescent state of HSCs and suggest the existence of different pathways downstream of β-catenin activation.

### 3.2. The Distinct Roles of CBP and p300

To generate a transcriptionally active complex, β-catenin must recruit either of the two Kat3 transcriptional coactivators CBP or p300 (adenovirus early region 1A (E1A)-binding protein; ~300 kDa) that are highly homologous to histone Kat3 acetyltranferases, as well as other components of the basal transcription apparatus [53]. Recent studies showed that CBP and p300 interact with hundreds of proteins in their roles as master regulators of transcription. Due to their high homology, these two coactivators have long been considered mostly redundant; however, accumulating evidence indicates that CBP and p300 are not redundant, but rather play definitive and unique roles both in vitro and in vivo [54]. Additionally, although analyses of transcription-factor-binding sites suggest that CBP and p300 share many common binding partners, activating protein (AP)-1 and serum-response factor appear to be more prominent interactors with CBP-specific sequences, whereas sites targeted by AP-2 and the transcription factor specificity protein 1 (SP1) are enriched with p300-specific target sequences [54]. CBP/β-catenin-mediated transcription is critical for proliferation/non-differentiation, whereas p300/β-catenin-mediated transcription initiates differentiation [30,55]. Therefore, specific inhibitors of CBP/β-catenin interaction have been developed to modulate the various effects mediated by CBP/β-catenin. 

### 3.3. Inhibitors of CBP/β-Catenin Interaction

#### 3.3.1. ICG-001

ICG-001 is a first-generation inhibitor of CBP/β-catenin interaction that binds to CBP but not to the related transcriptional coactivator p300, thereby specifically disrupting the interaction of CBP with β-catenin. ICG-001 was originally developed for cancer therapy, with ICG-001 treatment reported to selectively induce apoptosis in colon carcinoma cells but not in normal colonic epithelial cells [56]. Because ICG-001 selectively interacts with CBP, treatment with ICG-001 reduces the mRNA and protein expression of survivin, a member of the inhibitor of apoptosis gene family, and cyclin D1, which are downstream targets of CBP/β-catenin [17,18]. Effects of ICG-001 treatment on fibrosis have also been reported. After stimulation with TGF-β, mouse fibroblasts and human HSCs show increased mRNA levels of *Wnt3a*, *Wnt10*, *LRP6*, *αSMA*, and *collagen I*, with these increases inhibited by ICG-001 treatment [57]. Additionally, ICG-001 inhibits β-catenin signaling and attenuates bleomycin-induced lung fibrosis in mice while protecting the epithelium, with simultaneous administration of ICG-001 and bleomycin preventing pulmonary fibrosis, and late-stage administration effectively reversing fibrosis and significantly improving the survival rates of the animals [18]. In a mouse renal fibrosis model initiated by unilateral ureteral obstruction, ICG-001 treatment ameliorated interstitial fibrosis and suppressed the renal expression of fibronectin, collagen I, collagen III, αSMA, plasminogen activator inhibitor-1, fibroblast-specific protein-1, Snail1, and Snail2, with late-stage administration of ICG-001 also effectively attenuating fibrotic lesions in the obstructive nephropathy [19]. Moreover, in mice injected with CCl_4_, ICG-001 treatment significantly attenuated collagen accumulation and HSC activation [57]. Interestingly, ICG-001 treatment also drastically inhibited macrophage infiltration, intrahepatic inflammation, and angiogenesis, with this study demonstrating that C-X-C motif chemokine ligand 12, which is secreted by activated HSCs in a Wnt-dependent manner, activates macrophages, and promotes angiogenesis, was strongly suppressed both in vitro and in vivo following ICG-001 administration [57].

#### 3.3.2. PRI-724

PRI-724 is a second-generation CBP/β-catenin inhibitor developed by Prism Pharma [21] and exhibits antifibrotic effects in the liver according to animal models [21,58,59]. HCV transgenic (HCV-Tg) mice, in which HCV proteins are persistently expressed in the liver using the Cre/loxP switching system, develop liver fibrosis associated with liver injury [59]. Similar to humans, these mice subsequently develop steatosis, liver fibrosis, and hepatocellular carcinoma, suggesting their suitability as models for human HCV-related liver diseases [60]. A study using this model showed that PRI-724 treatment significantly attenuated increases in the area of collagen fibrils [61], as well as increases in hepatic hydroxyproline. These results clearly indicated that CBP/β-catenin signaling was activated in HCV-protein-expressing livers, and that inhibition of CBP/β-catenin by PRI-724 was effective at counteracting HCV-induced liver fibrosis. Moreover, PRI-724 treatment improved histological abnormalities in the hepatocyte-plate arrangement and morphology found in HCV-Tg mice without reducing HCV core-protein expression [61]. Additionally, this study reported that levels of αSMA, a marker of HSC activation, were higher in HCV-Tg mice as compared with those in control mice; however, these levels were attenuated by PRI-724 treatment. Similarly, immunohistochemistry results revealed that the number of αSMA-expressing cells increased in HCV-Tg mice relative to that in control mice, and that this induction was attenuated by PRI-724 treatment. These findings suggest that PRI-724 inhibits HSC activation and collagen production. In addition to reduced HSC activation, changes in the MMP/TIMP balance in the liver following PRI-724 treatment (*Mmp-8* mRNA levels were elevated, whereas those of *Timp-1* decreased) were also considered a mechanism underlying the observed antifibrotic effects of the drug in the HCV-Tg mice [61]. Furthermore, fluorescence-activated cell-sorting analysis of intrahepatic leukocytes from the HCV-Tg mice administered PRI-724 showed an increased number of Kupffer cells, neutrophils, Ly-6C^high^ monocytes, and Ly-6C^low^ monocytes, with immunohistochemical analysis revealing MMP-8 production in macrophages and neutrophils in the liver [61].

Additionally, PRI-724 treatment reduced CCl_4_-induced liver fibrosis in mice [21]. In CCl_4_-induced liver fibrosis, *S100a4* expression, which is controlled by CBP/β-catenin, increased in non-parenchymal cells, suggesting that CBP/β-catenin-mediated signals were activated in the liver. Moreover, PRI-724 treatment reduced liver fibrosis and *S100a4* expression without affecting serum alanine aminotransferase (ALT) levels, suggesting that the antifibrotic effects of PRI-724 are not due to the reduction of hepatocellular damage. Treatment with C-82, an active metabolite of PRI-724, inhibited the activation of isolated HSCs accompanied by suppression of the mRNA expression of *collagen I*, *αSMA* and *Timp-1*, as well as the protein expression of cyclin D, αSMA, and Ki67. Microarray analysis revealed that C-82 treatment abrogated the expression of a majority of genes upregulated in activated HSCs, whereas removal of C-82 rapidly restored gene expression associated with HSC activation. Furthermore, CCl_4_ treatment increased F4/80^+^CD11b^+^ and Ly6C^low^CD11b^+^ macrophages, which are responsible for fibrosis resolution [62], with these levels sustained during the fibrosis-resolution period, even after stopping CCl_4_ treatment. Administration of PRI-724 during the resolution period accelerated fibrosis resolution accompanied by induction of *Mmp-2*, *-8*, and *-9* mRNA expression in intrahepatic leukocytes, as well as the expression of MMP-8 protein in the liver [21]. These findings implicated CBP/β-catenin signaling in liver fibrosis and showed that PRI-724 treatment reduced liver fibrosis by both inhibition of HSC activation and increased resolution of inflammation by macrophages [21].

**Figure 3:**
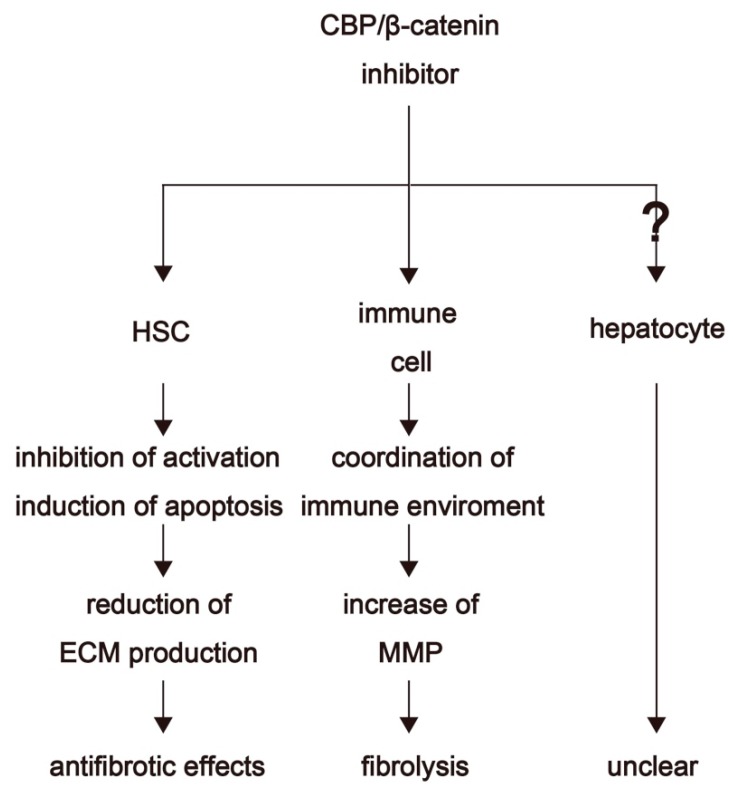
The effects of CBP/β-catenin inhibitors. CBP/β-catenin inhibitors exert known effects on HSCs and immune cells, but their precise effects in hepatocytes are still unclear.

The antifibrotic effects of PRI-724 have also been investigated in a nonalcoholic steatohepatitis (NASH) mouse model [58]. Treatment with a high-fat diet plus d-galactosamine induces liver fibrosis accompanied by hepatocyte apoptosis without hepatocellular damage (normal alanin aminotransferase (ALT) levels are maintained) in mice. The induction of hepatocyte apoptosis is crucial for liver fibrosis, as inhibition of hepatocyte apoptosis by treatment with antimicrobials, TNF-α knockdown, or overexpression of constitutively active inhibitor of nuclear factor kappa-B kinase (IKK) 2 results in reduced liver fibrosis. Both apoptosis and fibrosis were inhibited in NASH mice following PRI-724 treatment. Additionally, hepatocyte-specific CBP-knockout mice showed reduced liver fibrosis, as well as reduced hepatocyte apoptosis. Liver fibrosis also decreased in mice in which CBP was specifically knocked out in collagen-producing cells [58]. These results implicated CBP/β-catenin signaling in hepatocyte apoptosis and HSC activation during fibrosis formation in a NASH model. Furthermore, these findings support CBP/β-catenin inhibitors as potential regulators of the fibrotic liver microenvironment and suggest their potential for development as therapeutic drugs (Figure 3).

## 4. Therapeutic Options for Fibrosis Treatment through Inhibition of CBP/β-Catenin Interaction

### 4.1. Clinical Trials

**Figure 4:**
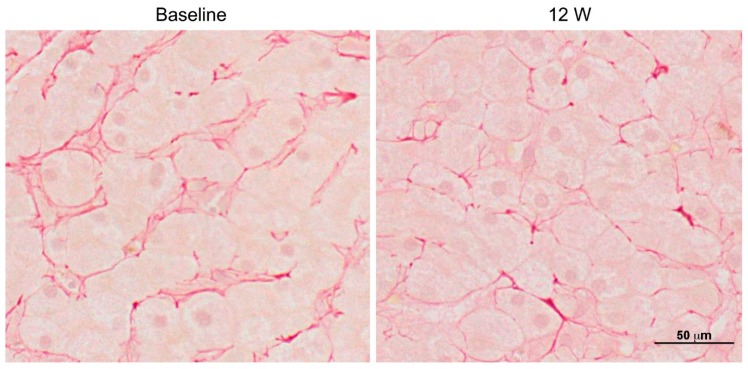
Histopathology of hepatic lobules stained with Sirius Red (400×). Liver biopsy sample from patient C2-03 (40 mg/m^2^/day PRI-724 in a CP class B cohort) at 12-weeks post-PRI-724 treatment. Scale bar = 50 μm.

Clinical trials using PRI-724 have been performed for advanced myeloid malignancies (49 participants) [63], advanced solid tumors (23 participants) [64], and advanced pancreatic cancer (20 participants) [65]. Recently, a single-center, open-label, phase I clinical trial investigating the safety and tolerance of PRI-724 in patients with HCV cirrhosis classified as Child-Pugh (CP) class A or B was completed (14 participants). Results showed that PRI-724 was generally well-tolerated, with most adverse events being grade 1 or 2. Most observed adverse events of PRI-724 administration were related to reaction at the injection site (64.3%) and gastrointestinal symptoms (nausea: 28.6%; vomiting: 14.3%; and constipation: 14.3%). Additionally, PRI-724 treatment was associated with histological improvement (a ≥2-point reduction in histologic activity index score) in three of the 12 patients; however, two showed deterioration by 2 points. Furthermore, histological analysis with silver and Sirius Red staining showed that PRI-724 reduced fibrosis (especially reticular fibrosis) in hepatic lobules in a dose-dependent manner (Figure 4) [66]. Intravenous injection of PRI-724 in 12 patients with HCV cirrhosis at doses of 10 mg/m^2^/day and 40 mg/m^2^/day appeared to be safe, provided that the plasma was exposed to the drug in a dose-dependent manner. The overall findings showed that PRI-724 resulted in improved liver histology and CP scores in several patients [ClinicalTrials.gov (no. NCT02195440)]. Because previous clinical studies using PRI-724 have only been conducted with small patient cohorts, further studies with larger sample sizes are required to confirm the safety and efficacy of the drug in patients with cirrhosis. A phase I/IIa clinical trial of PRI-724 for patients with liver cirrhosis has been initiated (study start date: 24 July 2018).

## 5. Conclusions

The effects and mechanisms of CBP/β-catenin inhibitors against liver fibrosis have been investigated in several studies. ICG-001 has shown promising antifibrotic effects in vitro and in vivo in animal models; however, it has not been tested extensively in clinical trials for its effects on hepatic fibrosis. PRI-724 shows antifibrotic efficacy in various murine liver fibrosis models, such as HCV-Tg models, models induced by CCl_4_ or bile-duct ligation, and NASH models. Phase I clinical trials of PRI-724 have demonstrated its safety, tolerability, and preliminary efficacy against HCV-induced cirrhosis. Moreover, PRI-724 shows great potential as a liver fibrosis therapeutic agent, although its impact on hepatocytes and other cells remains unclear. Liver fibrosis can be caused by various stimuli; therefore, further studies and clinical trials are required to investigate whether inhibition of CBP/β-catenin interaction would be a suitable antifibrotic strategy, irrespective of the cause of chronic liver damage.

## Abbreviations

ALT	Alanine aminotransferase
CBP	cAMP response element binding protein
ECM	Extracellular matrix
HCV	Hepatitis C virus
HSC	Hepatic stellate cell
DAMPs	Damage associated molecular patterns
ROS	Reactive oxygen species
CCL	C–C chemokine ligand
TGF	Transforming growth factor
αSMA	Smooth muscle actin
MMP	Matrix metalloproteinase
TIMP	Tissue inhibitors of metalloproteinase
LGR	Leucine-rich repeat-containing G protein-coupled receptor
CK1α	Casein kinase 1α
AP	Activating protein
LRP	Lipoprotein receptor
FZD	Frizzled
TCF	T cell factor
CCl_4_	Carbon tetrachloride
Tg	Transgenic
CP	Child-Pugh
